# Differential Gene Expression Patterns in Chicken Cardiomyocytes during Hydrogen Peroxide-Induced Apoptosis

**DOI:** 10.1371/journal.pone.0147950

**Published:** 2016-01-25

**Authors:** Chunyun Wan, Jinmei Xiang, Youwen Li, Dingzong Guo

**Affiliations:** 1 College of Veterinary Medicine, Huazhong Agricultural University, Wuhan, Hubei, People's Republic of China; 2 College of Animal Science, Yangtze University, Jingzhou, Hubei, People's Republic of China; 3 Hubei Vocational College Of Bio-Technology, Wuhan, Hubei, People's Republic of China; University of Nebraska-Lincoln, UNITED STATES

## Abstract

Hydrogen peroxide (H_2_O_2_) is both an exogenous and endogenous cytotoxic agent that can reliably induce apoptosis in numerous cell types for studies on apoptosis signaling pathways. However, little is known of these apoptotic processes in myocardial cells of chicken, a species prone to progressive heart failure. Sequencing of mRNA transcripts (RNA-Seq) allows for the identification of differentially expressed genes under various physiological and pathological conditions to elucidate the molecular pathways involved, including cellular responses to exogenous and endogenous toxins. We used RNA-seq to examine genes differentially expressed during H_2_O_2_-induced apoptosis in primary cultures of embryonic chicken cardiomyocytes. Following control or H_2_O_2_ treatment, RNA was extracted and sequencing performed to identify novel transcripts up- or downregulated in the H_2_O_2_ treatment group and construct protein−protein interaction networks. Of the 19,268 known and 2,160 novel transcripts identified in both control and H_2_O_2_ treatment groups, 4,650 showed significant differential expression. Among them, 55.63% were upregulated and 44.37% downregulated. Initiation of apoptosis by H_2_O_2_ was associated with upregulation of *caspase-8*, *caspase-9*, and *caspase-3*, and downregulation of anti-apoptotic genes *API5* and *TRIA1*. Many other differentially expressed genes were associated with metabolic pathways (including ‘Fatty acid metabolism’, ‘Alanine, aspartate, and glutamate metabolism’, and ‘Biosynthesis of unsaturated fatty acids’) and cell signaling pathways (including ‘*PPAR* signaling pathway’, ‘Adipocytokine signaling pathway’, ‘*TGF-beta* signaling pathway’, ‘*MAPK* signaling pathway’, and ‘*p53* signaling pathway’). In chicken cardiomyocytes, H_2_O_2_ alters the expression of numerous genes linked to cell signaling and metabolism as well as genes directly associated with apoptosis. In particular, H_2_O_2_ also affects the biosynthesis and processing of proteins and unsaturated fatty acids. These results highlight the value of RNA-seq for revealing unexpected molecular contributors to oxidative stress responses, thereby identifying novel potential therapeutic targets.

## Introduction

Cell apoptosis was first described in 1972 [1] and soon thereafter implicated in myocardial cell death associated with heart failure [2]. Hydrogen peroxide (H_2_O_2_) has well known cytotoxic effects. It is not only a common exogenous toxin, but is produced endogenously (e.g., by superoxide dismutase), which can lead to cellular apoptosis. Thus, it is widely used to induce apoptosis in toxicology research [3−5]. Myocardial cells are terminally differentiated; if these cells undergo apoptosis, they are not regenerated, leading to a progressive reduction in overall heart function and possible heart failure [2]. Thus, describing the mechanisms of apoptosis in cardiomyocytes is critical for understanding the pathogenesis of heart failure and for developing ameliorative treatments. Cardiomyocyte apoptosis and heart failure are common in chickens. Many strains of rapidly growing chickens are particularly susceptible to cardiomyocyte apoptosis and progressive of rapidly growing chickens are susceptible to heart disease, including heart failures to see if etnclude context-aheart failure as a result of diseases such as broiler pulmonary hypertension syndrome [6].

The sequencing of mRNA transcripts (termed RNA sequencing or RNA-Seq) is a maturing technology now widely used for the identification of differentially expressed genes, both known and without prior annotations [7]. While RNA-seq has been conducted to examine the mechanisms of resistance to *Campylobacter jejuni* colonization in chickens [8], it has not been applied to study apoptotic mechanisms in chicken myocardial cells. We induced apoptosis in chicken myocardial cells using H_2_O_2_ [5, 9] and identified differentially expressed genes by 100-bp paired-end reads using the Illumina HiSeq 2000 platform. In the late stage chicken embryo, heart development is nearly complete, and the number of myocardial cells rarely increases. Thus, primary cells isolated at this stage can exhibit the signaling responses of mature cardiomyocytes [10–12].

The aims of this study are threefold: (1) to identify the molecular signaling pathways involved in chicken cardiomyocyte apoptosis and repression of apoptosis, (2) to describe other changes in gene expression associated with the cytotoxicity of hydrogen peroxide, and (3) to evaluate the potential of RNA-seq for aims (1) and (2).

## Materials and Methods

### Ethics statement

This study was approved by the Animal Care and Use Committee of Hubei Province, China. All animal procedures were performed according to the guidelines developed by China’s Council on Animal Care.

### Isolation of chicken primary embryonic cardiomyocytes and induction of apoptosis

Monolayer cultures of embryonic chicken cardiomyocytes were prepared by the methods of DeHaan [13] with some modifications. Briefly, White Leghorn eggs were obtained from Beijing Merial Vital Laboratory Animal Technology (Beijing, China). At embryonic day 14 (E14), embryos were removed and decapitated in a Petri dish filled with Medium 199/EBSS (HyClone, Logan, Utah, USA) supplemented with 3% fetal bovine serum (FBS, Gibco, Grand Island, New York, USA). Ventricular tissues were isolated, pooled, and treated with 0.05% trypsin-EDTA to obtain a cell suspension as described [14]. We used the differential attachment technique to obtain high purity cells after 0.5 h of incubation. Cells were incubated in growth medium (Medium 199/EBSS containing 10% FBS) at 37°C under a 5% CO_2_ atmosphere. Cultures were washed three times at 8, 24, and 48 h to remove dead and dying cells. The serum concentration in the medium was then changed from growth (10%) to maintenance (2%) conditions, and incubation was continued for 36 h. The cells were then divided into two groups: a control group and an experimental group treated with 0.2 mM H_2_O_2_ for 10 h. The H_2_O_2_ dose and exposure time were determined by prior testing and by referencing previous studies [3, 4, 15]. The degree of apoptosis was estimated by DAPI staining. The control group was treated in the same way but with omission of H_2_O_2_. All individual treatments were repeated twice; replicates were named _1 and _2, respectively (e.g., H_1 and H_2). The RNA sample obtained from each replicate was bi-directionally sequenced, for four sequencing results per sample (named accordingly as H_1_1, H_1_2, H_2_1, and H_2_2).

### RNA sample collection and preparation

Total RNA was extracted using standard protocols (TRIzol, Invitrogen, CA, USA) and treated with DNase to remove any potential genomic DNA contamination. The quality of RNA was monitored by electrophoresis on 1% agarose gels. RNA purity and concentration were checked using a NanoPhotometer spectrophotometer (Implen, CA, USA) and Qubit RNA Assay Kit in a Qubit 2.0 Fluorometer (Life Technologies, CA, USA). RNA integrity was assessed using the Bioanalyzer 2100 system (Agilent Technologies, CA, USA).

### Library preparation for transcriptome sequencing

After total RNA extraction, mRNA was purified using poly-T oligo-attached magnetic beads [8]. Briefly, 3 μg of RNA was used as the input for each RNA sample preparation. Sequencing libraries were generated using a NEBNext Ultra RNA Library Prep Kit (Illumina, NEB, USA) following the manufacturer’s instructions. Index codes were assigned to attribute the sequences to each sample.

Clustering of the index-coded samples was performed on a cBot Cluster Generation System (Illumina) according to the manufacturer’s instructions. After cluster generation, the library preparations were sequenced on an Illumina HiSeq 2000 platform, and 100-bp paired-end reads were generated [16].

### Sequencing quality control and reads mapping to the reference genome

After sequencing, raw data in fastq format were first processed through in-house perl scripts. In this step, clean data were obtained by removing low-quality reads and reads containing the adapter sequence or poly-N. At the same time, Q20, Q30, and the GC content of the clean data were calculated. All subsequent analyses were based on these clean datasets.

Reference genome and gene model annotation files were directly downloaded from the Genome website (http://www.ncbi.nlm.nih.gov/genome/111?project_id=10808). An index of the reference genome was built using Bowtie v2.0.6, and paired-end clean reads were aligned to the reference genome using TopHat v2.0.9 [16].

### Quantification of gene expression levels

The reads mapped to each gene were counted by HTSeq v0.5.4p3. The RPKM (reads per kilobase of the exon model per million mapped reads) of each gene was then calculated based on gene length and read counts mapped. This method considers the effects of sequencing depth and gene length for the read counts and is currently the most reliable method for estimating gene expression levels [14].

### Alternative splicing and differential expression analysis

Alternative splicing was determined by Cufflinks 2.1.1 and ASprofile 1.0 software. Differential expression analysis was performed using the DESeq R package (1.10.1). DESeq provides statistical methods for determining differential expression in digital gene expression data using a model based on the negative binomial distribution. The resulting P-values were adjusted using Benjamini and Hochberg’s approach for controlling the false discovery rate; that is, genes with an adjusted P < 0.05 by DESeq were classified as differentially expressed. Corrected P values of 0.005 and log2 (fold change) values of 1 were set as the threshold for significant differential expression.

### GO and KEGG enrichment analysis of differentially expressed genes

After functional annotation, genes were further classified by Gene Ontology (GO) assignments. GO enrichment analysis of differentially expressed genes was performed using the GOseq R package, in which gene length bias was corrected. GO terms with corrected P < 0.05 were considered significantly enriched in differentially expressed genes [17].

The Kyoto Encyclopedia of Genes and Genomes (KEGG) is a database resource for the hierarchal categorization of genes and gene groups identified from genome sequencing and other high-throughput methods (http://www.genome.jp/kegg/). We used KOBAS software to test the statistical enrichment of differentially expressed genes in KEGG pathways [18, 19].

### Novel transcript prediction and alternative splicing analysis

The Cufflinks v2.1.1 Reference Annotation Based Transcript (RABT) assembly method was used to construct and identify both known and novel transcripts from the TopHat alignment results. Alternative splicing events were classified into 12 basic types by ASprofile v1.0. The number of AS events was estimated separately in each sample.

## Results

### Induction of apoptosis in chicken primary embryonic cardiomyocytes

We obtained high-purity myocardial cells from chicken embryonic ventricle. Isolated cells beat rhythmically and were immunopositive for α-actin. Hydrogen peroxide (0.2 mM for 10 h) induced substantial apoptosis as determined by DAPI staining (S1 Fig).

### Sequencing quality control

**Fig 1:**
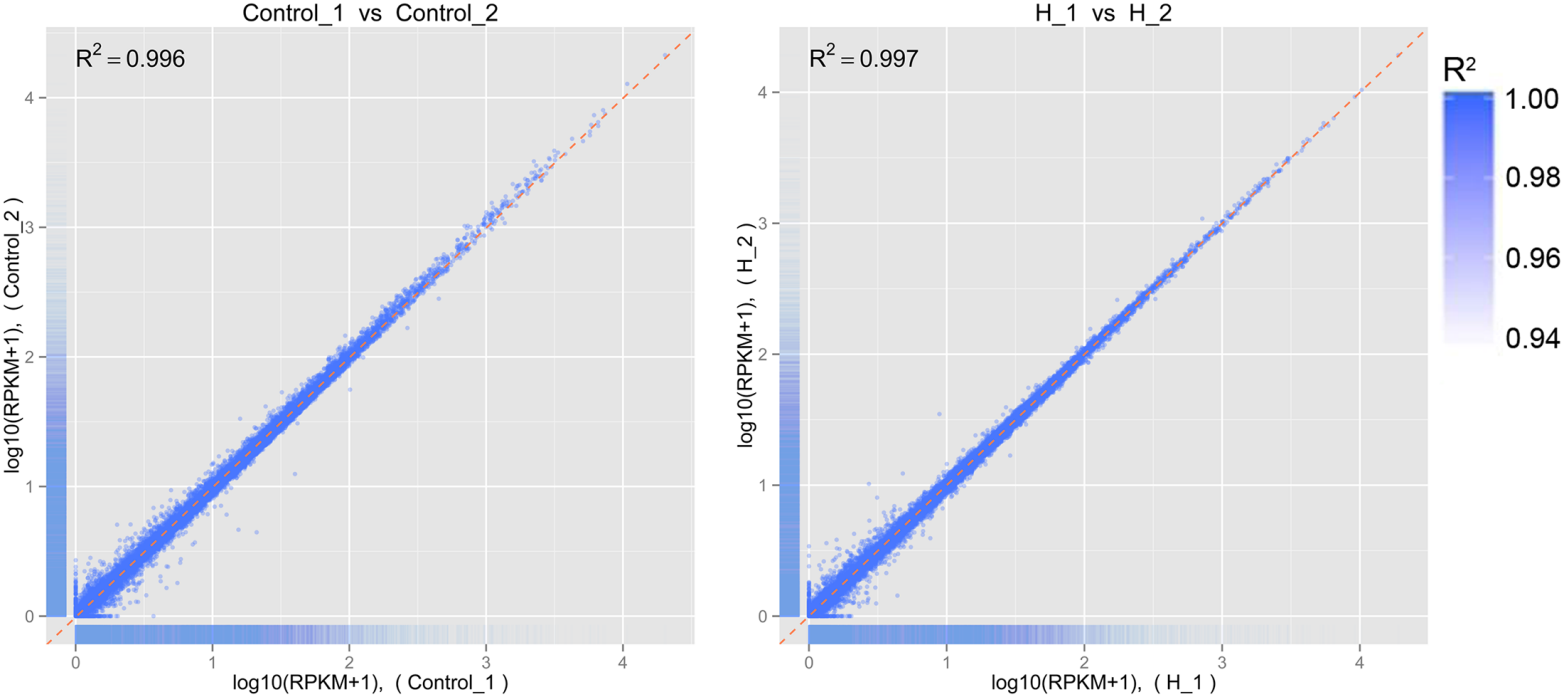
Correlations among technical replicates in each group. R^2^: square of the Pearson correlation coefficient.

After RNA sequencing, we assessed the quality of the data. The Q20, Q30, and GC content in the clean data were calculated (Table 1). Alignments between reads and the reference genome are presented in S1 Table. Replicates of each sample were sequenced, and the correlations between replicates are shown in Fig 1.

**Table 1:** Major characteristics of RNA-seq in the H_2_O_2_ (H) and Control group.

Sample name	Raw reads	Clean reads	Clean bases	Error rate(%)	Q20(%)^▲^	Q30(%)^▲^	GC content(%)^★^
Control_1_1	61286178	57687170	5.77G	0.03	97.15	91.50	53.26
Control_1_2	61286178	57687170	5.77G	0.04	96.03	89.60	53.32
Control_2_1	57289438	53642113	5.36G	0.04	97.03	91.19	54.04
Control_2_2	57289438	53642113	5.36G	0.04	95.75	88.93	54.09
H_1_1	59514696	56406015	5.64G	0.03	97.35	91.95	52.67
H_1_2	59514696	56406015	5.64G	0.04	96.30	90.12	52.73
H_2_1	62578501	59247377	5.92G	0.03	97.34	92.00	53.19
H_2_2	62578501	59247377	5.92G	0.04	96.39	90.34	53.24

^▲^Q20(%) and Q30(%) are the percentages of reads with Phred quality scores >20 and >30, respectively.

^★^ GC content(%) is G+C bases as a percentage of total bassese.

### Alternative splicing and differential expression analysis

**Fig 2:**
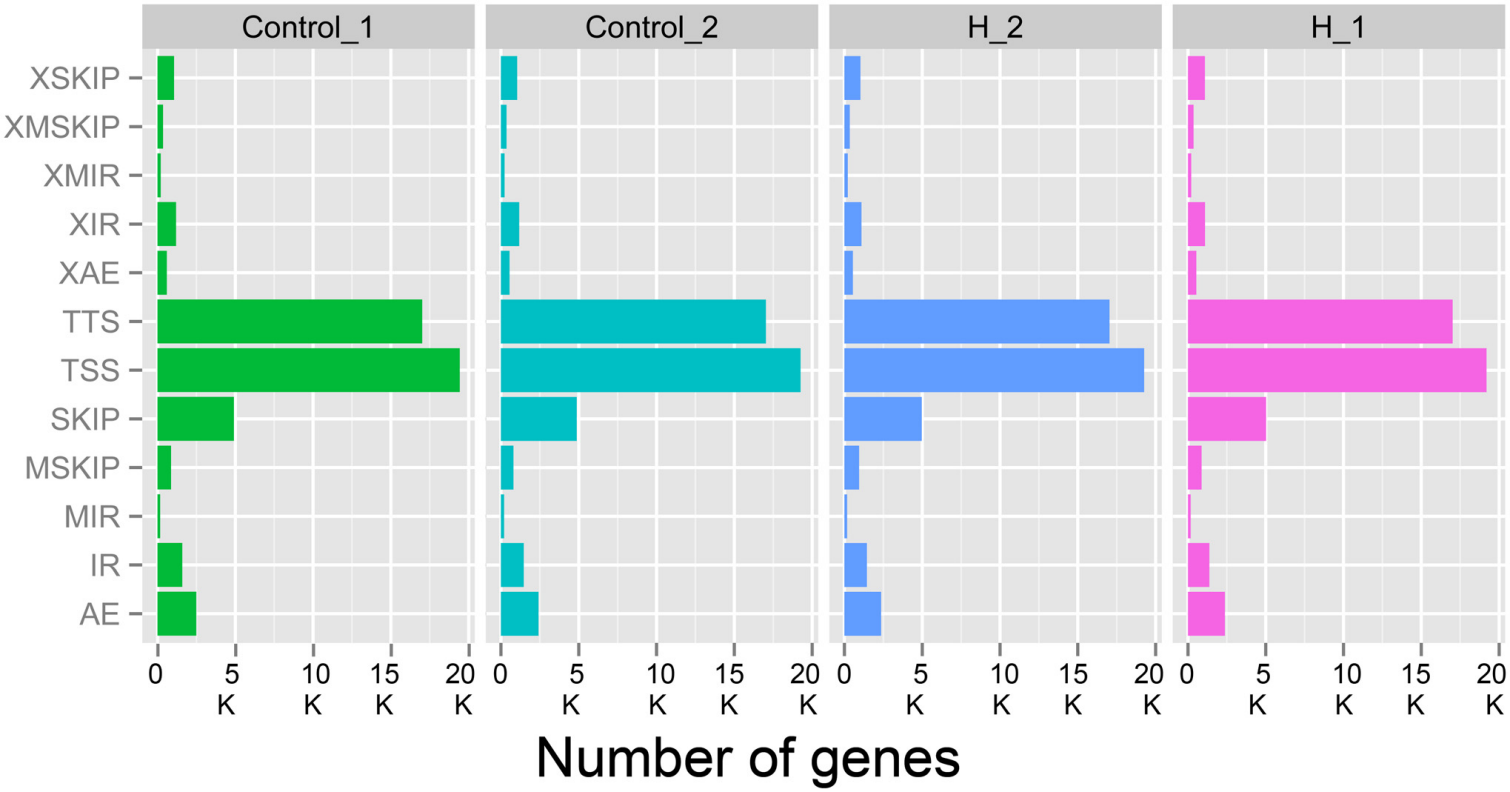
Statistics of the different alternative splicing events. The abbreviations indicate the different types of alternative splicing events as follows: SKIP: skipped exon; XSKIP: approximate SKIP; MSKIP: multi-exon SKIP; XMSKIP: approximate MSKIP; IR: intron retention; MIR: multi-IR; XMIR: approximate MIR; XIR: approximate IR; AE: alternative exon ends; XAE: approximate AE; TSS: alternative 5' first exon; TTS: alternative 3' last exon.

Alternative splicing (AS) analysis was performed using Cufflinks 2.1.1 and ASprofile 1.0. The alternative splicing event statistics are shown in Fig 2. The most common AS types were TSS (alternative 5′ first exon) and TTS (alternative 3′ last exon). We found some genes with more than five AS types.

**Fig 3:**
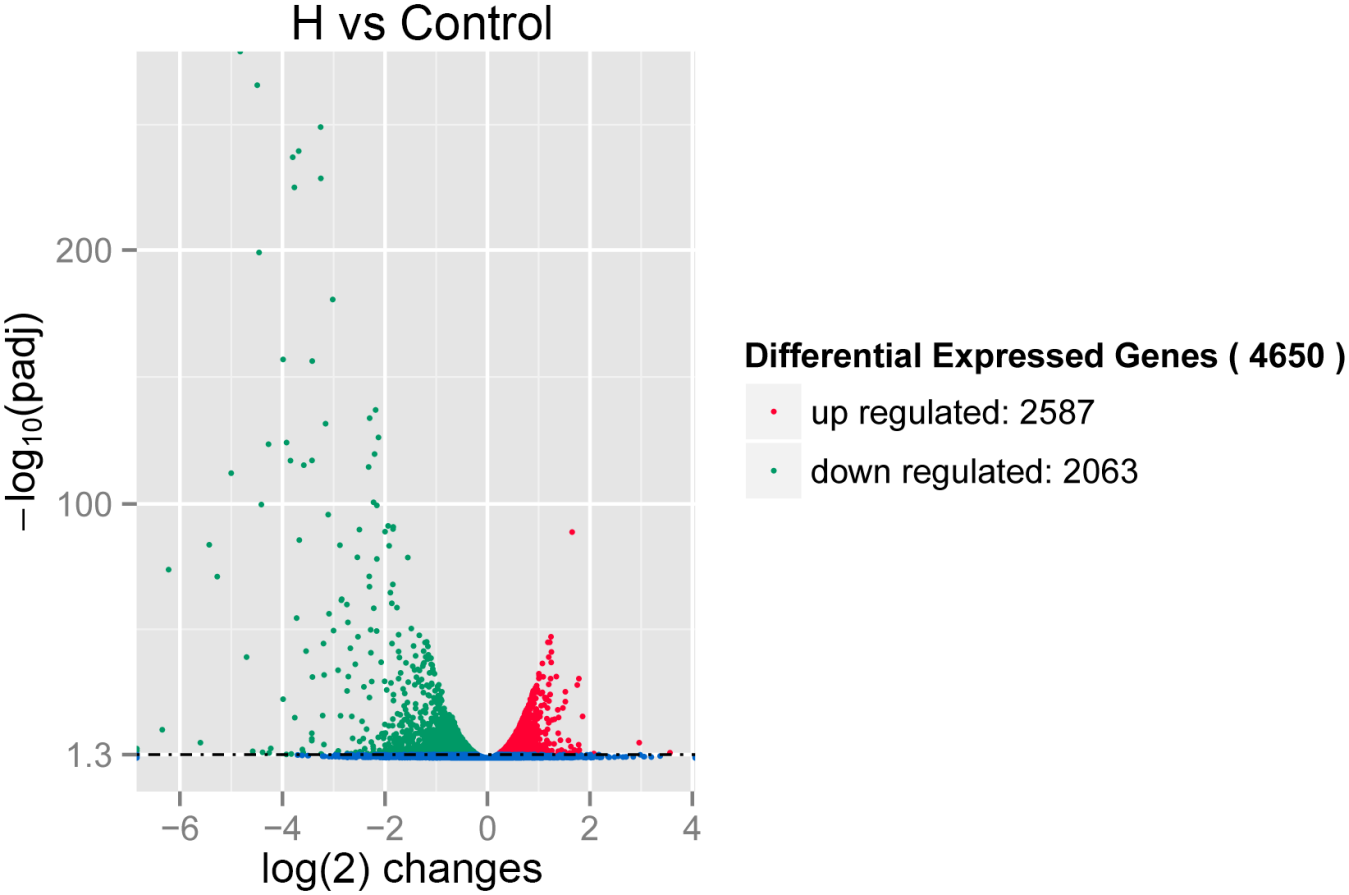
Volcano plot of differentially expressed genes. Each point represents one gene; red color indicates upregulation and green indicates downregulation.

To assess global transcriptional changes associated with apoptosis induction, we applied previously described methods [14] to identify differentially expressed genes from the normalized data. The results showed that 4,650 genes were significantly differentially expressed between control and H_2_O_2_-treated cultures. A volcano plot of differential gene expression (Fig 3) shows that 55.63% of all differentially expressed genes were upregulated and 44.37% downregulated.

### Validation of differential gene expression data by qPCR

**Fig 4:**
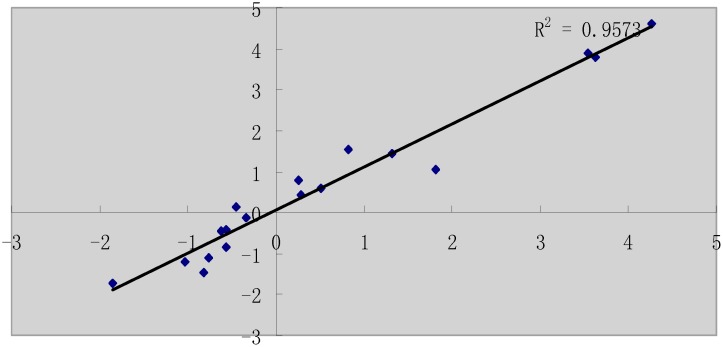
Validation of the RNA-Seq approach using qPCR. Eighteen genes that were differentially expressed between the H and control groups (10 upregulated and 8 downregulated) were selected. Their levels of expression were quantified by qRT-PCR analysis. The log2 changes in expression for the qRT-PCR and RNA-Seq data were closely correlated (r^2^ = 0.9573; p<0.01), confirming the accuracy of the RNA-Seq approach for quantification.

To validate the differentially expressed genes identified by sequencing, we selected 18 genes for qPCR confirmation, including 8 downregulated genes (*AMPK*, *EGLN1*, *FGF10*, *FOXO3*, *GHOX4*.*7*, *TGFBR*, *NF-κB*, *and MAPK*) and 10 upregulated genes (*CASP8*, *CASP9*, *CASP3*, *BAK1*, *TNFRSF1A*, *Bcl*, *Bcl2*, *CytC*, *P53*, *and XIAP*). The primers for the qPCR assays are shown in S2 Table. For the selected gene population, there was a strong correlation between RNA-Seq and qPCR results (r^2^ = 0.9573) (Fig 4), confirming the reliability of differential expression analysis using RNA-Seq.

### GO and KEGG enrichment analyses of differentially expressed genes

**Fig 5:**
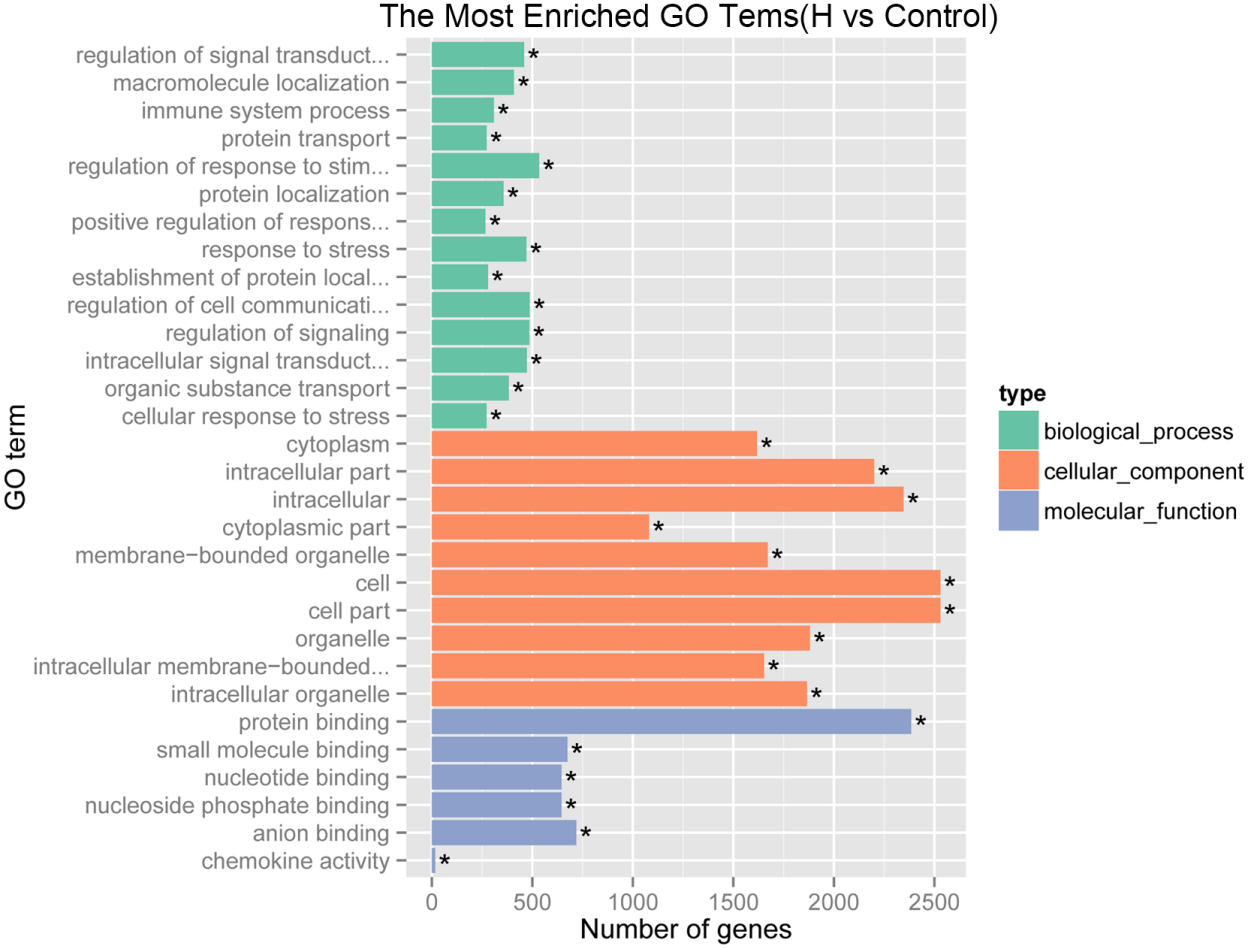
Results of GO enrichment analyses, including biological process, cellular component, and molecular function.

GO enrichment analysis of differentially expressed genes was performed using the GOseq R package. GO terms with corrected P < 0.05 were considered significantly enriched in differentially expressed genes. Genes were categorized into 10,563 GO terms within three domains: ‘biological process’, ‘cellular component’, and ‘molecular function’ (Fig 5). In the ‘biological process’ domain, the GO terms with highest enrichment of differentially expressed genes were ‘regulation of response to stimulus’, ‘response to stress’, and ‘macromolecule localization’. In the cell ‘component domain’, terms with highest enrichment were ‘intracellular’, ‘cell’, and ‘cell part’. We also observed a high percentage of differentially expressed genes assigned to ‘intracellular signal transduction’, ‘regulation of signal transduction’, and ‘regulation of cell communication’. Alternatively, few genes were assigned to terms such as ‘establishment of nucleus localization’ and ‘fibronectin binding’.

**Fig 6:**
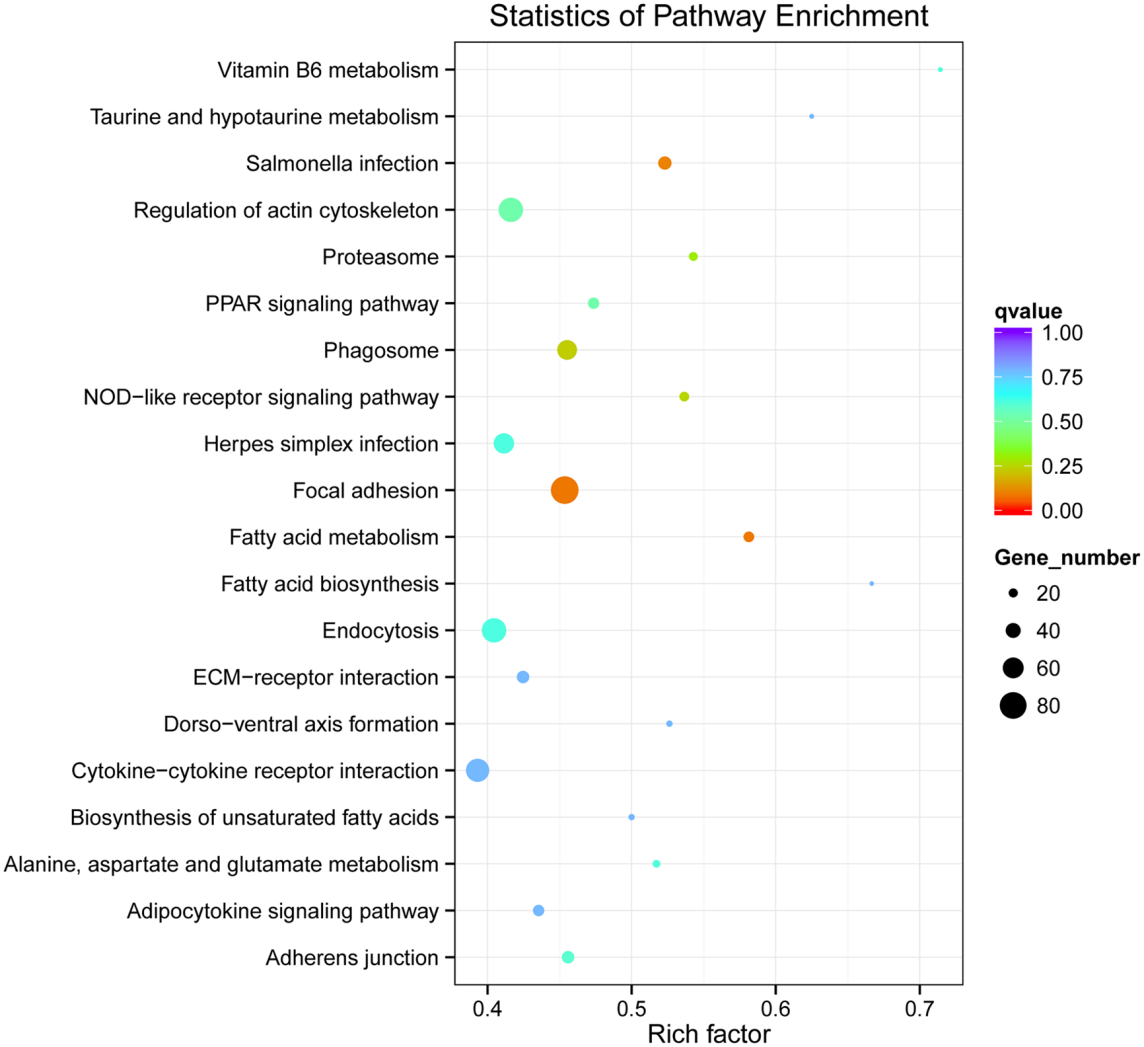
Statistics of KEGG enrichment. The size of each point indicates the number of differentially expressed genes in that pathway, and the color of each point indicates the q-value (adjusted p value). A higher enrichment factor indicates a greater degree of enrichment.

We analyzed the biological pathways that were active in our samples. Genes were mapped to the reference pathways in the KEGG and assigned to 152 KEGG pathways (S1 File). A few pathways, including the cytokine-cytokine receptor interaction pathway, *MAPK* signaling pathway, metabolic pathways, and regulation of the actin cytoskeleton pathway, contained large numbers of differentially expressed genes, while others, such as sulfur metabolism, contained only a few (Fig 6).

## Discussion

A previous gene expression profiling study of infracted myocardial tissue with signs of oxidative stress reported over a thousand differentially expressed genes, of which hundreds were sensitive to administration of antioxidants [20]. Furthermore, gene chip studies have shown that direct application of cytotoxic concentrations of H_2_O_2_ or other oxidants results in the up- or downregulation of hundreds to thousands of known protein-encoding genes [21, 22]. In present study, the first using RNA-Seq to examine gene expression changes during H_2_O_2_-induced oxidative stress and apoptosis in chicken myocardial cells, we identified over 6000 differentially expressed genes. This vast number reflects not only proteins directly related to apoptosis, but also myriad protein expression cascades involved in mitochondrial function, cell cycle regulation, DNA repair, protein translation and turnover, and antioxidant responses [23−26]. Genes transiently induced in the early phase of oxidative stress include a variety of redox-sensitive transcription factors that in turn activate multiple downstream targets [21, 27]. Thus, these numbers may be underestimates as expression levels of many genes likely return to baseline before profiling [22]. Transient early expression also explains the variation in expression profiles among cell types and oxidative stress induction paradigms. Despite this variation, the assembled annotated transcriptomes reported in these studies provide a valuable resource for understanding myocardial cell apoptosis and other cellular changes induced by oxidative stress. In contrast to many previous studies, however, the RNA-seq profiling presented here revealed over 2000 novel genes, underscoring the utility of this expression profiling method for identifying genes not previously implicated in cytotoxic responses by other profiling techniques.

### The classification of novel genes

After sequencing, a total of 2,160 novel genes were found. We blasted these genes and investigated open reading frames (ORFs) using ORF Finder [http://www.ncbi.nlm.nih.gov/projects/gorf/]. Most novel genes were noncoding RNAs, including many long noncoding RNAs (LncRNAs), suggesting that LncRNAs are involved in the regulation of apoptosis and other signaling processes evoked by H_2_O_2_. Indeed, multiple LncRNAs have been implicated in chronic cardiovascular diseases, myocardial cell proliferation, and cell death [28, 29].

### Screening pathways related to hydrogen peroxide toxicity

The differentially expressed genes were mapped to reference pathways in the KEGG and assigned to 152 KEGG pathways, including many metabolic and signaling pathways not directly related to apoptosis. For instance, within the metabolism domain, H_2_O_2_ altered expression of genes involved in ‘Fatty acid metabolism’, ‘Alanine, aspartate and glutamate metabolism’, ‘Biosynthesis of unsaturated fatty acids’, ‘Protein processing in endoplasmic reticulum’, ‘Vitamin B6 metabolism’, ‘Pyruvate metabolism’, and ‘Pantothenate and CoA biosynthesis’. We conclude that H_2_O_2_ has broad effects on cell metabolism, mainly on fatty acid, alanine, aspartate, and glutamate metabolism. It also affects ‘cell biosynthesis’, particularly protein processing and fatty acid synthesis, possibly as part of the repair response for mitigating H_2_O_2_-induced oxidative damage to proteins and lipid membranes.

Exposure to H_2_O_2_ also activated the ‘*PPAR* signaling pathway’, ‘Adipocytokine signaling pathway’, ‘*TGF-beta* signaling pathway’, ‘Toll-like receptor signaling pathway’, ‘*MAPK* signaling pathway’, ‘*p53* signaling pathway’, and ‘*Wnt* signaling pathway’. Most of these pathways are known regulators of apoptosis, such as ‘*MAPK* signaling pathway’ and ‘*p53* signaling pathway’. The *Wnt* signaling isoform *WNT5A* enhanced H_2_O_2_-resistance in fibroblasts [25], so *Wnt* pathway activation may represent a cytoprotective response. In contrast, *NF-kB* is a well known stress response gene linked to cell death, at least when stress is severe. Glucocorticoid inhibition of *NF-kB* in macrophages blocked H_2_O_2_-induced apoptosis [30]. Alternatively, *MAPKs* are broadly cytoprotective. The balance between *NF-kB* and *MAPK* signaling may determine cell fate (apoptosis vs. survival) under oxidative stress [31].

In addition, H_2_O_2_ altered the expression of genes associated with ‘Regulation of actin cytoskeleton’ and ‘cell cycle’, possible reflecting the changes in morphology and proliferation rate in response to H_2_O_2_ exposure. Several previous studies have reported modulation of cell cycle regulators and reduced proliferation concomitant with the antioxidant response [24, 25].

GO enrichment analysis indicated that many differentially expressed genes are associated with ‘cellular response to chemical stimulus’, ‘response to stress’, ‘regulation of signal transduction’, ‘cell migration’, ‘response to oxygen levels’, and ‘protein metabolic process’, in line with the KEGG analysis.

### Differential expression of apoptosis regulators

We examined the expression levels of *caspase-2*, *caspase-8*, *caspase-9*, and *caspase-10* [32–34] and of the effector caspases *caspase-3*, *caspase-6*, and *caspase-7* [35, 36]. Our results showed that apoptosis initiation predominantly relies on *caspase-8* and *caspase-9* upregulation, leading to *caspase-3* activation, so H_2_O_2_-induced apoptosis in chicken cardiomyocytes depends on the canonical apoptosis pathway. We also examined the expression of several apoptosis repressors, including *bcl-2*, *CFLAR*, *CAAP1*, *IAP*, *BFAR*, *TRIA1*, *API5*, and *CAPP1* [37−41]. By comparing expression levels in the apoptosis group with those in the control group, we found that the repression of apoptosis may rely on *API5* and *TRIA1*. In addition, GO enrichment analysis identified two novel genes that may be involved in apoptosis repression. However, we also found that other anti-apoptosis genes, such as *IAP* and *BFAR*, were significantly downregulated following H_2_O_2_ treatment. Other differentially expressed genes are less direct regulators of apoptosis. Products of the downregulated *EGLN* gene family, hypoxia-inducible factor prolyl hydrolases, are ubiquitination-dependent suppressors of hypoxia-inducible factors (*HIFs*), transcription factors that induce a variety of cytoprotective responses during oxidative stress [42]. Downregulation of *EGLN* genes and concomitant enhancement of *HIF* activity could serve to protect against H_2_O_2_-induced oxidative stress. However, H_2_O_2_ also downregulated the forkhead family transcription factor *FOXO3*, which is known to upregulate antioxidant enzymes, including catalase [43]. Thus, H_2_O_2_ activated both cell death and cytoprotective pathways (also including the X-linked inhibitor of apoptosis protein (*XIAP*).

### Differential expression of cell cycle regulators

In addition to apoptosis-associated genes, H_2_O_2_ altered expression of genes associated with cell cycle regulation and DNA damage. Genes regulating the cell cycle, such as *CDK4-6*, *Bax*, *TP53*, *CDC2*, and *DK1* [44–46] were all significantly differentially expressed, suggesting that H_2_O_2_ may reduce the cardiomyocyte population in the embryo by suppressing proliferation.

### RNA-seq is suitable for using in toxicology research

While RNA-seq is frequently used to screen differentially expressed genes and novel genes, it has rarely been employed in toxicology research. Our work demonstrates the utility of RNA-Seq for screening of toxicological responses in cells and tissues. Further studies to assess the applicability of RNA-seq to in vivo toxicology are warranted.

## Conclusion

Cytotoxic effects of hydrogen peroxide have been reported in many studies, but the full panoply of differentially expressed genes had not been examined by RNA-seq. We used RNA-Seq to identify genes differentially expressed in cardiomyocytes undergoing H_2_O_2_-induced apoptosis. Sequencing identified 19,268 known genes and 2,160 novel genes, of which 4,650 were significantly differentially expressed between control and H_2_O_2_-treated cells. Apoptosis was associated with upregulation of *caspase-8*, *caspase-9*, and *caspase-3*, and downregulation of anti-apoptotic genes *API5* and *TRIA1*. However, H_2_O_2_ exposure was also associated with changes in many genes associated with metabolic and signaling pathways. Hydrogen peroxide appears to have myriad effects on cell biosynthesis (protein processing, fatty acid synthesis), morphology, and the cell cycle. Furthermore, this study demonstrates the potential of RNA-seq for molecular toxicology research.

## Supporting Information

S1 Fig Culture and identification of primary cardiomyocyte. (A) Untreated primary cardiomyocytes (100×). (B) Primary cardiomyocytes stained by DAPI after H_2_O_2_-induced apoptosis (100×), (C) Immunocytochemical staining showing abundant α-actin (400×) in chicken cardiomyocytes. (D) Control fibroblasts showing no α-actin immunoreactivity (400×).
(TIF)

S1 File KEGG enrichment of differential expressed genes. (XLS)

S1 Table Alignment between reads and reference genome. (DOC)

S2 Table Primers used for qRT-PCR analysis. (DOC)
