# Supplementary figures and images for: Differential Gene Expression Patterns in Chicken Cardiomyocytes during Hydrogen Peroxide-Induced Apoptosis
Source: PLoS One. 2016 Jan 25;11(1):e0147950. doi: 10.1371/journal.pone.0147950 (PMC4726744; doi:10.1371/journal.pone.0147950)

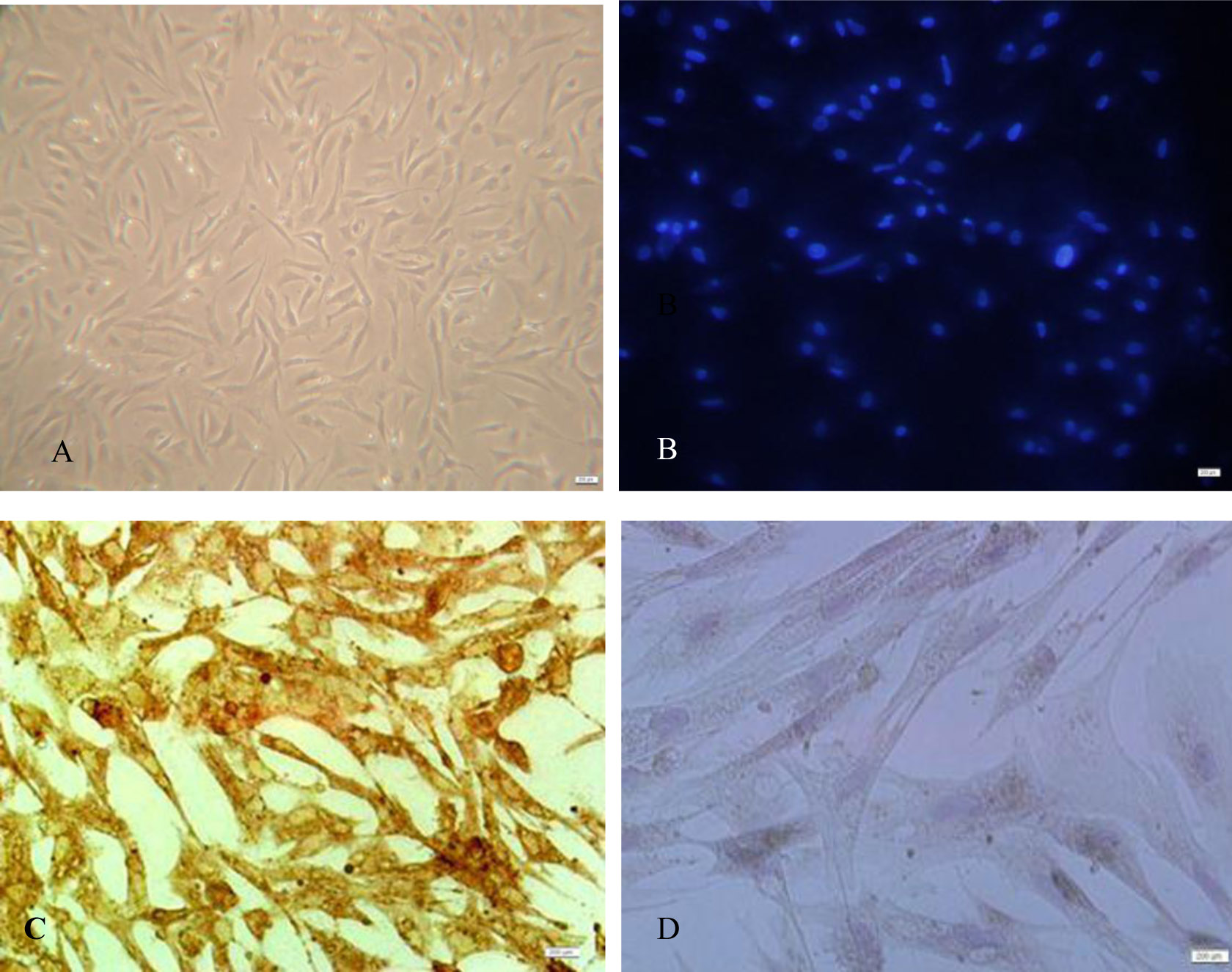

Supplement: S1 Fig — (A) Untreated primary cardiomyocytes (100×). (B) Primary cardiomyocytes stained by DAPI after H2O2-induced apoptosis (100×), (C) Immunocytochemical staining showing abundant α-actin (400×) in chicken cardiomyocytes. (D) Control fibroblasts showing no α-actin immunoreactivity (400×). (TIF) [file pone.0147950.s001.tif]
