# Supplementary material for: Differential Gene Expression Patterns in Chicken Cardiomyocytes during Hydrogen Peroxide-Induced Apoptosis
Source: PLoS One. 2016 Jan 25;11(1):e0147950. doi: 10.1371/journal.pone.0147950 (PMC4726744; doi:10.1371/journal.pone.0147950)
Supplement: S1 Table — (DOC) [file pone.0147950.s003.doc]

**S1 Table. Alignment between reads and reference genome**

| Sample name | Control_1 | Control_2 | H_1 | H_2 |
| --- | --- | --- | --- | --- |
| Total reads | 115374340 | 107284226 | 112812030 | 118494754 |
| Total mapped | 91503521 (79.31%) | 83841695 (78.15%) | 91373115 (81%) | 95047224 (80.21%) |
| Multiple mapped | 1600343 (1.39%) | 1402762 (1.31%) | 1547274 (1.37%) | 1649778 (1.39%) |
| Uniquely mapped | 89903178  (77.92%) | 82438933  (76.84%) | 89825841  (79.62%) | 93397446 (78.82%) |
| Read-1^▲^ | 45188675 (39.17%) | 41417088 (38.61%) | 45095629 (39.97%) | 46868458 (39.55%) |
| Read-2^▲^ | 44714503 (38.76%) | 41021845  (38.24%) | 44730212 (39.65%) | 46528988 (39.27%) |
| Reads map to '+'^★^ | 44938933 (38.95%) | 41207084  (38.41%) | 44904973  (39.81%) | 46691372 (39.4%) |
| Reads map to '-'^★^ | 44964245 (38.97%) | 41231849  (38.43%) | 44920868  (39.82%) | 46706074 (39.42%) |
| Non-splice reads | 55928960  (48.48%) | 50563133  (47.13%) | 54865544  (48.63%) | 57109004 (48.2%) |
| Splice reads | 33974218  (29.45%) | 31875800  (29.71%) | 34960297  (30.99%) | 36288442 (30.62%) |

^▲^Read-1, Read-2: repeat sequencing of each sample.

^★^Reads map to '+'，Reads map to '-'：reads to positive and negative chain of Genome.
