# Supplementary material for: Differential Gene Expression Patterns in Chicken Cardiomyocytes during Hydrogen Peroxide-Induced Apoptosis
Source: PLoS One. 2016 Jan 25;11(1):e0147950. doi: 10.1371/journal.pone.0147950 (PMC4726744; doi:10.1371/journal.pone.0147950)
Supplement: S2 Table — (DOC) [file pone.0147950.s004.doc]

**S2 Table. Primers used for the qPCR analysis**

| Gene | Forward | Reverse |
| --- | --- | --- |
| *AMPK* | TGCAGCACTGAGACCTGGAT | CAGTTGCTGCGGATGAAGTC |
| *EGLN1* | CGAGGCCATCAACTTCCTTC | TCCACATGACGCACATACCC |
| *FGF10* | AGACACGTGCGGAGCTACAAT | ACCTTGCCGTTCTTCTCGAT |
| *FOXO3* | ACAAACCGTGCACTGTGGAGT | TGGAGCTTCTCTGCATCATCC |
| *GHOX4.7* | TTGATCAGAACATTCCCGTCC | GGAAATTAGCCTCAGCCTGGT |
| *TGFBR* | AGACTGCAGCGATGTTTGTGC | GGCAGCAATTCTGTGTTGTGG |
| *NF-κB* | GGACTTAAAATGGCAGGAGAG | GCTGTTCGTAGTGGTAAGTCTG |
| *MAPK* | GCGGCTCCGCTAAAATGCCG | GGGGTGAGGTTCTGGTAGCGC |
| *CASP8* | ACTGACATGGACTGAAGGA | TTACAGTGACGTGCTCCA |
| *CASP9* | TCAGACATCGTATCCTCCA | AAGTCACAGCAGGGACA |
| *CASP3* | GATGCTGCAAGTGTCAGA | ATCGCCATGGCTTAGCA |
| *BAK1* | GCCCTGCTGGGTTTCGGTAA | AATTCGGTGACGTAGCGGGC |
| *TNFRSF1A* | CCTGCTCCTCATCATTGTGT | TGATCCATGTACTCCTCTCC |
| *Bcl* | GCTTTGAGCAGGTAGTGA | CAAGTACGTGGTCATCCAA |
| *Bcl-2* | TGAGCAGAGGTCACGTA | CACACTGTGGAACAGCA |
| *CytC* | CCTGTCCTGGTGCATGATG | TACTCTGATCCAGCTCTGCCTGAA |
| *P53* | ACCTGCACTTACTCCCCGGT | TCTTATAGACGGCCACGGCG |
| *XIAP* | GGGCATCACATAGGAGCGCA | TCCTTCCACTCTTGCAATCC |
| *β-actin* | CTGATGGTCAGGTCATCACCATT | ACCCAAGAAAGATGGCTGGA |
